# Nematode Species Identification—Current Status, Challenges and Future Perspectives for Cyathostomins

**DOI:** 10.3389/fcimb.2017.00283

**Published:** 2017-06-28

**Authors:** Christina M. Bredtmann, Jürgen Krücken, Jayaseelan Murugaiyan, Tetiana Kuzmina, Georg von Samson-Himmelstjerna

**Affiliations:** ^1^Department of Veterinary Medicine, Institute for Parasitology and Tropical Veterinary Medicine, Freie Universität BerlinBerlin, Germany; ^2^Department of Veterinary Medicine, Institute for Animal Hygiene and Environmental Health, Freie Universität BerlinBerlin, Germany; ^3^Department of Parasitology, I.I. Schmalhausen Institute of ZoologyKyiv, Ukraine

**Keywords:** cyathostomins, nematodes, diagnostic, PCR, MALDI-TOF MS

## Abstract

Human and animal health is globally affected by a variety of parasitic helminths. The impact of co-infections and development of anthelmintic resistance requires improved diagnostic tools, especially for parasitic nematodes e.g., to identify resistant species or attribute pathological effects to individual species or particular species combinations. In horses, co-infection with cyathostomins is rather a rule than an exception with typically 5 to 15 species (out of more than 40 described) per individual host. In cyathostomins, reliable morphological species differentiation is currently limited to adults and requires highly specialized expertize while precise morphological identification of eggs and early stage larvae is impossible. The situation is further complicated by a questionable validity of some cyathostomins while others might actually represent cryptic species complexes. Several molecular methods using different target sequences were established to overcome these limitations. For adult worms, PCR followed by sequencing of mitochondrial genes or external or internal ribosomal RNA spacers is suitable to genetically confirm morphological identifications. The most commonly used method to differentiate eggs or larvae is the reverse-line-blot hybridization assay. However, both methods suffer from the fact that target sequences are not available for many species or even that GenBank® entries are unreliable regarding the cyathostomin species. Recent advances in proteomic tools for identification of metazoans including insects and nematodes of the genus *Trichinella* will be evaluated for suitability to diagnose cyathostomins. Future research should focus on the comparative analysis of morphological, molecular and proteomic data from the same cyathostomin specimen to optimize tools for species-specific identification.

## Introduction

Parasitic helminths globally affect human and animal health and can be of zoonotic relevance (e.g., *Ascaris* spp.). In equines, the most important intestinal nematodes belong to the family Strongylidae and are comprised of two subfamilies: The Strongylinae encompassing 14 species in 5 genera (*Strongylus, Oesophagodontus, Triodontophorus, Bidentostomum*, and *Craterostomum*), and the Cyathostominae encompassing currently 50 valid species in 14 genera (*Caballonema, Coronocyclus, Cyathostomum, Cylicocyclus, Cylicodontophorus, Cylicostephanus, Cylindropharynx, Gyalocephalus, Hsiungia, Parapoteriostomum, Petrovinema, Poteriostomum, Scrjabinodentus, Tridentoinfundibulum*) (Lichtenfels et al., 2008), in contrast to previous publications listing 51 or 52 species in 13 genera (Lichtenfels, 1975; Lichtenfels et al., 2002). In the literature, the term “small strongyles” has either been coined to include only the Cyathostominae or all equine strongylidae except the genus *Strongylus*, which were designated “large strongyles” (Lyons et al., 1999). Although, still widely used, it is now recommended to avoid the terms small and large strongyles (Lichtenfels et al., 2002).

Since prevalence of the highly pathogenic *Strongylus* species declined after introduction of the macrocyclic lactones (Herd, 1990), the cyathostomins are currently recognized as the most important equine parasites because of (i) their up to 100% prevalence in equids (Lyons et al., 1999), (ii) numerous reports of anthelmintic resistance and (iii) their pathogenicity which becomes particularly manifest in cases of sometimes fatal larval cyathostominosis (Love et al., 1999). Anthelmintic resistance against benzimidazoles is highly prevalent worldwide and pyrantel resistance is also frequently observed whereas reduced efficacy of macrocylic lactones has rarely been reported (Kaplan, 2002; Kuzmina and Kharchenko, 2008; Von Samson-Himmelstjerna, 2012; Matthews, 2014; Nielsen et al., 2014).

Cyathostomins have a direct life-cycle with adults located in the lumen of caecum and colon, shedding eggs with the feces. First larvae (L1) hatch in the feces, molt twice to infectious third larvae (L3) which are ingested by equids. In the large intestine, L3 encyst inside the intestinal wall and may also undergo hypobiosis for months, before molting to fourth larvae (L4) (Corning, 2009). Synchronous excystation of large numbers of hypobiotic larvae potentially causes larval cyathostominosis characterized by severe inflammation leading to weight-loss, diarrhea, colic, or even death (Love et al., 1999).

Although, Cyathostomins are a threat to equine welfare and scientific efforts to address this problem are frequently undertaken, research is impaired by the lack of sufficient identification methods (Lichtenfels, 2008). This perspective addresses the different methods, their advantages and limitations and gives an outlook on possible future methods for nematode identification using the cyathostomins as paradigm.

## Samples and sampling

The first challenge for species identification is the availability of suitable specimens. While strongylid eggs can be easily collected from feces, they have virtually no diagnostically useful morphological features. Strongylid L3 can be obtained from eggs using different fecal cultures methods (Smyth, 1990). However, only some L3 can be identified to the genus level, and this requires a high experience level. Only for a few species *in vitro* culture to the L4 (Chapman et al., 1994; Brianti et al., 2009) has been described. Therefore, adult parasites must be collected from naturally infected hosts. In horses, only a few adult strongyles are occasionally shed with the feces but collection of adult nematodes from feces after anthelmintic treatment is possible (Osterman Lind et al., 2003; Kuzmina et al., 2005; Kuzmina and Kharchenko, 2008). However, the complete worm burden representing all species in the living horse will only be documented by examination of all feces over several days, which may be associated with degradation of worms leading to distorted results. A more exact and meaningful method is the collection of adult nematodes from the content of the horse intestine (Drudge and Lyons, 1977). The critical test method, which is described in detail by Drudge et al. (1963), is a combination of both, the fecal collection over a week and collection during necropsy. This method is widely used to study the effectiveness of anthelmintic compounds (e.g., Lyons et al., 2007, 2010). Due to the need of sacrificed or slaughtered horses, these methods are restricted to research. Thus, there is a great need to develop alternatives for precise nematode diagnosis for living horses. The immediate research aim is therefore the development of effective and specific non-invasive cyathostomin identification methods.

## Morphological identification

For more than 100 years (Molin, 1861; Loos, 1900), a large number of cyathostomin species has been morphologically described using 93 different names. In the meantime, several previously described species are considered synonyms (Lichtenfels et al., 1998) and currently 50 species are recognized as valid. Comprehensive identification keys summing up the descriptions were published (Lichtenfels, 1975; Tolliver, 2000; Lichtenfels et al., 2008).

**Figure 1:**
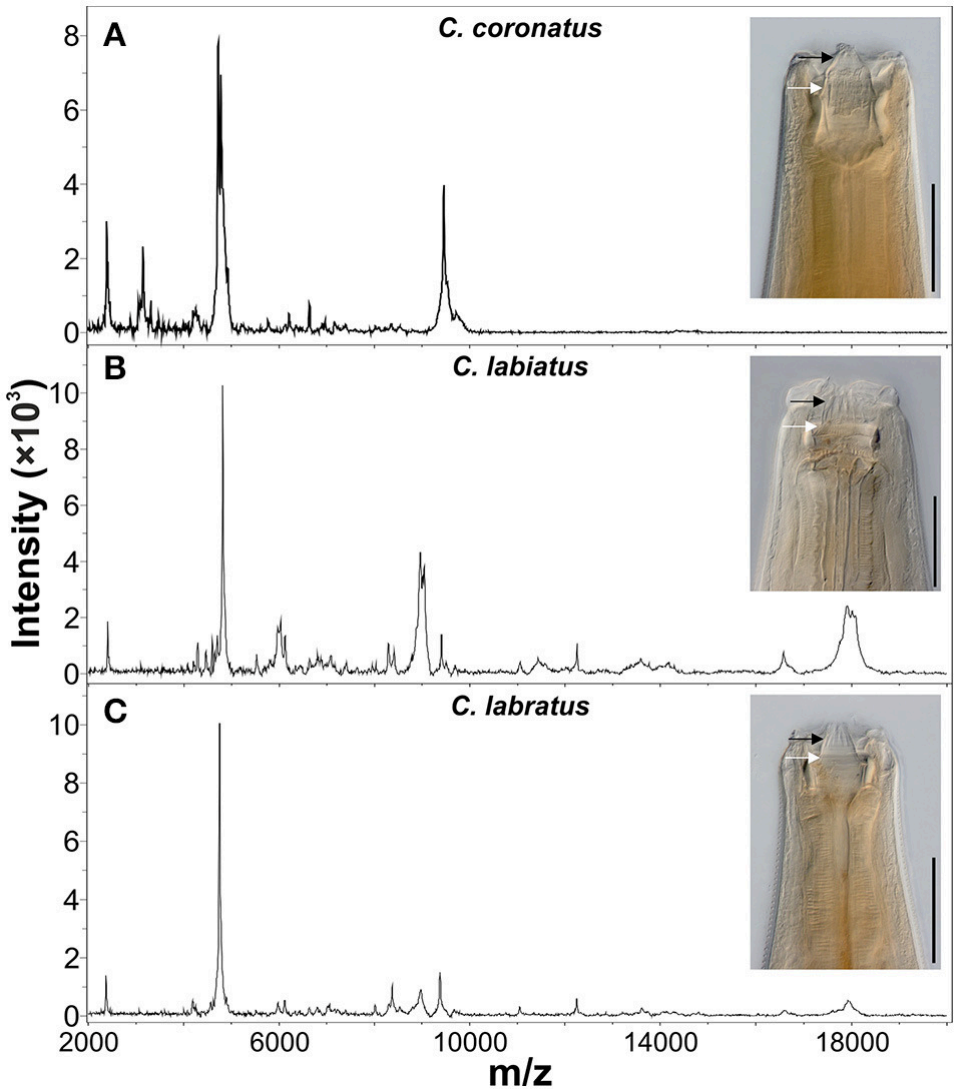
Comparison of morphological and proteomic species identification methods. Anterior ends and representative MALDI-TOF MS spectra of three cyathostomin species from the closely related species **(A)** *Coronocyclus coronatus*, **(B)** *Coronocyclus labiatus*, and **(C)** *Coronocyclus labratus* are shown. Scale bars represent 100 μm. The x-axes show mass charge ratios while y-axes represent arbitrary intensity units. Spectra were baseline subtracted and smoothed using default parameters in the flexAnalysis software (Bruker Daltonics). Specimen were cleared with lactophenol to improve visibility of structural features of the cuticle. External and internal leaf crown are indicated by black and white arrows, respectively.

Morphological identification of adult strongyles relies on careful examination of faint characters at the anterior end of the adult nematodes or of the reproductive system. These traits include the size and shape of buccal capsules, internal and external leaf crowns and its extra-chitinous support as illustrated in Figure 1 to point out that differences are very faint. Fine morphological structures of posterior end such as size and shape of the bursa, genital cone, gubernaculum, and spicules in males and shape of the tail, size and proportion of different parts of the reproductive system in females are also valuable for species differentiation (Lichtenfels, 1975; Dvojnos and Kharchenko, 1994; Lichtenfels et al., 2008). However, reliable morphological identification of adult cyathostomins can only be achieved following several years of intensive training and currently only few experts are available worldwide (Lichtenfels et al., 2008).

Whereas, adult cyathostomins can be discriminated, eggs, L1 and L2 cannot be differentiated from other nematodes of the family Strongylidae. Identification of L3 is possible for some genera such as *Strongylus, Triodontophorus, Gyalocephalus*, or *Poteriostomum* while most others can only be assigned to several cyathostomin larval types (Bevilaqua et al., 1993; Santos et al., 2016). The morphological features include qualitative and quantitative traits such as the number, arrangement and shape of intestinal/midgut cells, the length of the intestine and the length of the sheath tail.

## Molecular methods

To overcome the limitations of morphological identification, research has focused on molecular cyathostomin identification. These methods, once target-sequences are implemented correctly, can be applied independently of the nematode life-stage.

A target locus which proved to be useful in developing genetic markers for diagnostic and phylogenetic purposes is the ribosomal DNA (rDNA) (reviewed by Gasser and Newton, 2000; Chilton, 2004). Eukaryotic nuclear rDNA is organized in clusters of sometimes several hundred repeats. Coding sequences for 18S, 5.8S, and 28S rRNAs are interrupted by the first and second internal-transcribed spacers (ITS-1 and ITS-2) (Long and Dawid, 1980). The similarity of ITS sequences is higher within than among different species (Elder and Turner, 1995). This was also shown for strongyles, where the extent of intraspecific variation was low (0–0.3%) in comparison to interspecific differences (0.6–23.7% for the ITS-1 region, 1.3–56.3% for the ITS-2 region) (Hung et al., 1999b).

An early approach for molecular species identification based on the ITS-2 locus is the PCR-linked restriction fragment length polymorphism (PCR-RFLP) analysis, which was first used for differentiation of single eggs of the Strongylinae (Campbell et al., 1995) and was then applied to show that the morphologically very similar *Cylicocyclus ashworthi* and *Cylicocyclus nassatus* actually represent separate species (Hung et al., 1997). Another method is the PCR-linked single-strand-conformation-polymorphism technique (SSCP-PCR; Gasser and Monti, 1997), which allows the delineation of 14 strongyle species, including 9 cyathostomins, based on ITS-2 PCR products (Hung et al., 1999a). These methods rely on the DNA from individual worms or eggs and are thus associated with time-consuming procedures if it is desired to screen a representative subset of a strongyle community.

Species identification from mixed parasite DNA from fecal samples and/or copro-cultures was demonstrated after ITS-2 sequences for 28 strongyle species (including 22 cyathostomin species) were determined and species specific primers evaluated for four common species (Hung et al., 1999b). Although, this method theoretically allows species-specific research on pooled samples, it is limited to the identification of only few species.

The variability of the 26S-18S rDNA intergenic-spacer (IGS) was used for species differentiation of 16 cyathostomin species with a range of interspecies variation of 31–56% (Kaye et al., 1998). The obtained sequences were used to develop a PCR-ELISA for the identification of six common cyathostomin species (Hodgkinson et al., 2003) and a Reverse-Line-Blot-Assay (RLB) to simultaneously identify 13 strongyle species (Traversa et al., 2007). Both methods have been used to monitor the species composition before and after anthelmintic treatment (Hodgkinson et al., 2005; Čerňanská et al., 2009; Ionita et al., 2010; Traversa et al., 2010). Re-evaluation and validation of existing and new oligo-probes increased the number of species that can be identified with RLB to 18 (Cwiklinski et al., 2012). PCR-ELISA and RLB are qualitative methods detecting the presence or absence of the different species. A semi-quantitative approach applying replicates of pooled larvae was positively evaluated to enable screening of many cyathostomin populations in parallel (Kooyman et al., 2016).

Despite being recognized as “a less suitable target than ITS for quick diagnostic tests,” due to its high substitution rates and high possibility of intraspecific polymorphisms, the mitochondrial Cytochrome oxidase c subunit I (COI) is used for species differentiation and could indicate cryptic species (Blouin, 2002). Twenty two COI sequence haplotypes (overall 10.8% rate of intraspecific nucleotide difference) were found within *C. nassatus* using specimen from different hosts and geographic origins, while only little variation (0.0–0.6% differences) was seen in the ITS-2 sequences suggesting cryptic species within *C. nassatus* (Traversa et al., 2008) and maybe other cyathostomin morpho-species as well. Analysis of ITS-1 and ITS-2 sequences of *Cylicostephanus minutus* individuals showed 3.0 and 7.4% differences also indicating the presence of a cryptic species complex (Hung et al., 1999a). The combination of markers on questionable species appears useful to investigate the occurrence of cryptic species complexes.

Whereas the objective of research on cyathostomin species identification is on the one hand to improve the available diagnostic tools, it aims on the other hand to contribute to the understanding of the phylogenetic relationships between the different taxa. Therefore, three gene loci, the ITS-2, COI and 28S rRNA were compared for their phylogenetic usefulness in strongyles. It was encountered that the high level of substitution saturation renders COI unsuitable for phylogenetic analysis. The remaining loci, ITS-2 and 28S rRNA, both showed similar groupings of cyathostomins. Combining both loci resulted in a tree with improved bootstrapping support for the internal nodes (McDonnell et al., 2000) pointing towards the importance of the simultaneous application of different molecular markers. This can also be seen in a study analyzing ITS-1 and ITS-2 sequences of 30 strongyle nematode species, including 23 cyathostomin species that questions the widely accepted separation of Strongylinae and Cyathostominae and proposes a framework to systematically analyze future datasets of strongyle nematodes (Hung et al., 2000). Findings consistent with the latter phylogenetic analysis were shown in a study focusing on the genus *Cylicocyclus* which proposed a separation of *Cylicocyclus* in two clades but statistical support for this hypothesis was relatively weak (Bu et al., 2013).

## Serological methods

Larval cyathostominosis is caused by the simultaneous re-activation and emergence of high numbers of hypobiotic larvae (Love et al., 1999) causing severe pathology. Usually, no eggs are expelled due to absence of adults making coproscopic diagnosis unfeasible (Murphy and Love, 1997). This leads to the aim of pre-patent detection of cyathostomin infections using serology to be able to assess the risk of larval cyathostominosis based on the estimation of the mucosal cyathostomin worm burden.

One promising approach identified anti-larval IgG(T) serum antibody responses to two antigen complexes, only elicited by larvae, as potential markers for prepatent cyathostomin infections (Dowdall et al., 2002). Subsequent purification of native antigenic complexes from larvae resulted in higher IgG(T) signals in an ELISA and reduced the number of false positive responses (Dowdall et al., 2003). Further evidence for an immunodiagnostic potential of these markers is given by a study where the mucosal worm burden of naïve and infected horses was assessed and found to significantly correlate with the IgG(T) serum levels. Additionally, sera from horses with clinical suspicion of larval cyathostominosis had significantly increased antigen-specific IgG(T) levels (Dowdall et al., 2004). One antigenic complex could be identified as cyathostomin gut-associated larval antigen-1 (Cy-GALA-1) and allocated to the species *Cyathostomum pateratum* (McWilliam et al., 2010), followed by the characterization of the orthologous antigens of four additional common cyathostomin species. An ELISA was developed based on recombinant Cy-GALA proteins, which allows the detection of the immune response to cyatostomin larvae. Cross-reactivity to other parasites was not observed and is unlikely, because of the diversity of orthologous GALA sequences of non-cyathostomin species (Mitchell et al., 2016). In the absence of experimental single species infections, cross-reactivity between cyathostomin species is hard to evaluate and diagnostic tests should therefore include a panel of different Cy-GALA proteins to detect most larval cyathostomin infections.

If in future available for routine diagnosis this approach could be of clinical relevance and help ruling out or confirming a larval cyathostominosis in horses with unspecific symptoms of wasting or colic. However, due to the lack of species-specificity serological methods will not help gaining detailed knowledge on the role of individual species in larval cyathostominosis.

## Proteomics method

The proteome based matrix-assisted laser desorption/ionization time-of-flight mass spectrometry (MALDI-TOF MS profiling) species identification of microorganisms has already revolutionized diagnostic microbiology. Species identification is based on the molecular masses of proteins such as ribosomal and other abundant proteins. A small amount of microorganisms or crude extracted intact proteins is transferred to specially designed target plates and allowed to co-crystallize with an inert, UV absorbing matrix such as α-Cyano-4-hydroxycinnamic acid. A pulsed 337 nm laser beam irradiates the samples to form a dense ion plume. The resultant ions are accelerated through a vacuum tube to reach the detector and separated according to their charge/mass (*m/z*) ratio and the time of flight (TOF) for each is measured. The mass range *m/z* 2,000–20,000 is generally applied for species identification through pattern matching of the spectra peaks with that of a reference spectra database. The method is popular due to its cost-effectiveness, reliability and availability of specially designed linear MALDI machines equipped with software tools and reference databases. This method has been evaluated for a variety of microorganisms such as bacterial, fungal, and viral pathogens (Wieser et al., 2012; Clark et al., 2013). In the past two decades, this technique was established for rapid characterization of eukaryotic cell lines and for species differentiation of protozoan parasites (e.g., *Leishmania, Giardia*) and arthropods (e.g., mosquitoes, ticks, tsetse flies) (Hoppenheit et al., 2013; Singhal et al., 2016; Yssouf et al., 2016). Regarding nematodes, first diagnostic use of MALDI-TOF has been described to identify different races of *Ditylensus dipsaci* (Perera et al., 2009) and closely related species of root-knot and seed-gall nematodes (Perera et al., 2005; Ahmad et al., 2012). Despite these developments, extensive studies on the application of MALDI-TOF MS for rapid species identification for helminth have not been reported. Recently, MALDI-TOF MS was applied for rapid species identification of *Trichinella* spp. after adopting a simple formic-acid/acetonitrile extraction from pooled larvae and compilation of a reference database (Mayer-Scholl et al., 2016). This approach could also be extended to cyathostomin species identification. Preliminary data to evaluate the potential for MALDI-TOF MS for cyathostomins revealed distinct patterns for adult individuals of different species (Figure 1).

Of course, master-spectra libraries can only be generated with validated, correctly identified material. This requires that proteomic data are obtained from morphologically and molecularly identified individual specimen. For arthropods, this issue can be solved by using always e.g., a wing or leg for proteomic and any other body part for molecular analysis. For nematodes, which are not segmented, this is not trivial since no defined body parts can be reproducibly cut off at exactly the same position without altering the protein spectrum. Therefore, methods need to be developed that reliably allow to conduct both methods using exactly the same starting material, despite the fact that the protein extraction usually involves conditions that damage DNA. Nevertheless, it was possible to extract DNA of sufficient quality from the acetonitrile/formic acid insoluble material to successfully amplify and sequence the ITS-2 region for the three specimen shown in Figure 1. These were 100, 99, and 97% identical to Genbank accession numbers JN786951.2, JN786947.2, JN786949 respectively, which confirmed the morphological identification in each case.

Possible limitations could be the different spectra elicited by different development stages, as seen in tick species identification. However, despite changes of the overall MS protein profiles, the ticks could be classified correctly according to certain specific peaks (Karger et al., 2012). Characterization of species-specific peak patterns, independent of development stages, therefore needs to be part of future research to implement MALDI-TOF MS as a possible diagnostic tool.

## Conclusion

Different approaches have been used over the past decades to improve cyathostomin species delineation. All methods have their advantages and limitations (Table 1) and none is already fully satisfying for the research questions to be answered and all are far away from applicability in routine laboratory diagnosis. Comprehensive research on different aspects improving the discrimination of individual cyathostomin species, such as inclusion of several molecular markers and additional proteomic profiles could be of great help in the future. This should include the morphological identification together with the description of the genotype (molecular) and phenotype (proteomic) data in association with the currently accepted taxonomic classification. Ideally, morphological, molecular and proteomic data from the same individual should be used to take advantage of all three approaches to identify the complete species spectrum in the Cyathostominae and delineate their phylogenetic relationship.

**Table 1:** Comparison of methods for cyathostomin species identification.

**Identification method**		**Life stage**	**Usefulness and limitations**	**References**
**MORPHOLOGICAL IDENTIFICATION**				
		Eggs	No species differentiation possible	Lichtenfels, 1975, 2008; Dvojnos and Kharchenko, 1994; Tolliver, 2000; Lichtenfels et al., 2008; Kharchenko et al., 2009; Kornaś et al., 2009; Santos et al., 2016
		Larvae	L3 can be allocated to different larvae types, but not to individual species	
		Adults	Identification keys published but species identification is difficult for inexperienced workers	
**MOLECULAR METHODS**				
**Marker**	**Method**			
ITS-1 and ITS-2	PCR and sequencing	All	Species identification and phylogenetic analysis, identification of cryptic species Sometimes only small differences between closely related species Not applicable for mixed samples, isolation of DNA from individual specimen necessary	Campbell et al., 1995; Chilton et al., 1997; Hung et al., 1999a, 2000; Bu et al., 2013
	SSCP-PCR	All	Delineation of 14 Strongylida species (9 Cyathostomin species), Isolation of DNA from individual specimen necessary	Gasser and Monti, 1997
	PCR-RFLP	All	Distinction of Strongylinae eggs, Distinction of two Cyathstomin species (*C.ashworthi, C.nassatus*). Isolation of DNA from individual specimen necessary, established for larvae	Campbell et al., 1995; Hung et al., 1997
IGS	PCR-ELISA	All	Screening for 6 cyathostomin species in mixed samples possible. Established for eggs and larvae	Hodgkinson et al., 2003, 2005
	RLB	All	Differentiation of up to 18 common species, less time consuming and costly than other molecular methods, screening of strongyle population before and after anthelmintic treatment possible, mixed samples possible, but more viable for individual worms; semi-quantitative approach possible	Traversa et al., 2007; Ionita et al., 2010; Cwiklinski et al., 2012; Kooyman et al., 2016
COI	PCR and sequencing	All	Investigation of intraspecies genetic variability, identification of cryptic species, not applicable for mixed samples, isolation of DNA from individual specimen necessary	Hung et al., 1999a; Traversa et al., 2008
**SEROLOGICAL METHODS**				
Protein-based ELISA		Larvae	Pre-patent detection of four common cyathostomin species possible from serum, no cyathostomin species differentiation possible	Mitchell et al., 2016
**PROTEMICS METHOD**				
		Potentially all	Method is established for bacteria, fungi, several species of arthropods; only one study on nematodes (*Trichinella* spp.), protocols and reference spectra data base have to be established	Karger et al., 2012; Yssouf et al., 2013, 2016; Mayer-Scholl et al., 2016; Singhal et al., 2016;

## Author contributions

CB performed MALDI-TOF experiments and literature surveys. CB and JK drafted and edited the manuscript. TK contributed microphotographs. TK, JM, and GS contributed to writing of the manuscript. CB, JK, JM, and GS designed the general outline.

### Conflict of interest statement

The authors declare that the research was conducted in the absence of any commercial or financial relationships that could be construed as a potential conflict of interest. The reviewer MN declared a past co-authorship with one of the authors GvS to the handling Editor, who ensured that the process met the standards of a fair and objective review.

## References

[B1] Ahmad F. Gopal J. Wu H. (2012). Rapid and highly sensitive detection of single nematode via direct MALDI Mass Spectrometry. Talanta 93, 182–185. 10.1016/j.talanta.2012.02.009 22483896

[B2] Bevilaqua C. M. L. Rodrigues M. de L. Concordet D. (1993). Identification of infective larvae of some common nematode strongylids of horses. Rev. Med. Vet. 144, 989–995.

[B3] Blouin M. S. (2002). Molecular prospecting for cryptic species of nematodes: mitochondrial DNA versus internal transcribed spacer. Int. J. Parasitol. 32, 527–531. 10.1016/S0020-7519(01)00357-5 11943225

[B4] Brianti E. Giannetto S. Traversa D. Chirgwin S. R. Shakya K. Klei T. R. (2009). *In vitro* development of cyathostomin larvae from the third stage larvae to the fourth stage: morphologic characterization, effects of refrigeration, and species-specific patterns. Vet. Parasitol. 163, 348–356. 10.1016/j.vetpar.2009.04.029 19493623

[B5] Bu Y. Niu H. Zhang L. (2013). Phylogenetic analysis of the genus Cylicocyclus (Nematoda: Strongylidae) based on nuclear ribosomal sequence data. Acta Parasitol. 58, 167–173. 10.2478/s11686-013-0124-z 23666651

[B6] Campbell A. J. D. Gasser R. B. Chilton N. B. (1995). Differences in a Ribosomal DNA sequence of strongylus species allows identification of single eggs. Int. J. Parasitol. 25, 359–365. 10.1016/0020-7519(94)00116-6 7601594

[B7] Chapman M. R. Hutchinson G. W. Cenac M. J. Klei T. R. (1994). *In vitro* culture of equine strongylidae to the fourth larval stage in a cell-free medium. J. Parasitol. 80, 225–231. 10.2307/3283751 8158465

[B8] Chilton N. B. (2004). The use of nuclear ribosomal DNA markers for the identification of bursate nematodes (order Strongylida) and for the diagnosis of infections. Anim. Heal. Res. Rev. 5, 173–187. 10.1079/AHR200497 15984323

[B9] Chilton N. B. Gasser R. B. Beveridge I. (1997). Phylogenetic relationships of australian strongyloid nematodes inferred from ribosomal DNA sequence data. Int. J. Parasitol. 27, 1481–1494. 10.1016/S0020-7519(97)00134-3 9467733

[B10] Clark A. E. Kaleta E. J. Arora A. Wolk D. M. (2013). Matrix-Assisted laser desorption ionization-time of flight mass spectrometry: a fundamental shift in the routine practice of clinical microbiology. Clin. Microbiol. Rev. 26, 547–603. 10.1128/CMR.00072-12 23824373 PMC3719498

[B11] Corning S. (2009). Equine cyathostomins: a review of biology, clinical significance and therapy. Parasit. Vectors 2(Suppl. 2):S1. 10.1186/1756-3305-2-s2-s1 19778462 PMC2751837

[B12] Cwiklinski K. Kooyman F. N. J. Van Doorn D. C. K. Matthews J. B. Hodgkinson J. E. (2012). New insights into sequence variation in the IGS region of 21 cyathostomin species and the implication for molecular identification. Parasitology 139, 1063–1073. 10.1017/S0031182012000467 22717256

[B13] Dowdall S. M. J. Matthews J. B. Mair T. Murphy D. Love S. Proudman C. J. (2002). Antigen-specific IgG (T) responses in natural and experimental cyathostominae infection in horses. Vet. Parasitol. 106, 225–242. 10.1016/S0304-4017(02)00085-7 12062511

[B14] Dowdall S. M. J. Proudman C. J. Klei T. R. Mair T. Matthews J. B. (2004). Characterisation of IgG(T) serum antibody responses to two larval antigen complexes in horses naturally- or experimentally-infected with cyathostomins. Int. J. Parasitol. 34, 101–108. 10.1016/j.ijpara.2003.09.008 14711595

[B15] Dowdall S. M. J. Proudman C. J. Love S. Klei T. R. Matthews J. B. (2003). Purification and analyses of the specificity of two putative diagnostic antigens for larval cyathostomin infection in horses. Res. Vet. Sci. 75, 223–229. 10.1016/S0034-5288(03)00116-4 13129671

[B16] Čerňanská D. Paoletti B. Král'ová-Hromadová I. Iorio R. Čudeková P. Milillo P.. (2009). Application of a Reverse Line Blot hybridisation assay for the species-specific identification of cyathostomins (Nematoda, Strongylida) from benzimidazole-treated horses in the Slovak Republic. Vet. Parasitol. 160, 171–174. 10.1016/j.vetpar.2008.10.078 19042091

[B17] Drudge J. H. Lyons E. T. (1977). Methods in the evaluation of antiparasitic drugs in the horse. Am. J. Vet. Res. 38, 1581–1586. 337859

[B18] Drudge J. H. Szanto J. Wyant Z. N. Elam G. (1963). Critical tests of thiabendazole as an anthelmintic in the horse. Am. J. Vet. Res. 24, 1217–1222. 14081458

[B19] Dvojnos G. M. Kharchenko V. O. (1994). Strongylids of Wild and Domestic Horses (in Russian). Kiev: Naukova Dumka.

[B20] Elder J. F. J. Turner B. J. (1995). Concerted evolution of repetitive DNA sequences in eukaryotes. Q. Rev. Biol. 70, 297–320. 10.1086/419073 7568673

[B21] Gasser R. B. Monti J. R. (1997). Identification of parasitic nematodes by PCR-SSCP of ITS-2 rDNA. Mol. Cell. Probes 11, 201–209. 10.1006/mcpr.1997.0106 9232619

[B22] Gasser R. B. Newton S. E. (2000). Genomic and genetic research on bursate nematodes: significance, implications and prospects. Int. J. Parasitol. 30, 509–534. 10.1016/S0020-7519(00)00021-7 10731573

[B23] Herd R. P. (1990). The changing world of worms: the rise of the cyathostomes and the decline of strongylus vulgaris. Compend. Contin. Educ. Pract. Vet. 12, 732–736.

[B24] Hodgkinson J. E. Freeman K. L. Lichtenfels J. R. Palfreman S. Love S. Matthews J. B. (2005). Identification of strongyle eggs from anthelmintic-treated horses using a PCR-ELISA based on intergenic DNA sequences. Parasitol. Res. 95, 287–292. 10.1007/s00436-004-1289-z 15682337

[B25] Hodgkinson J. E. Lichtenfels J. R. Mair T. S. Cripps P. Freeman K. L. Ramsey Y. H.. (2003). A PCR-ELISA for the identification of cyathostomin fourth-stage larvae from clinical cases of larval cyathostominosis. Int. J. Parasitol. 33, 1427–1435. 10.1016/S0020-7519(03)00140-1 14527525

[B26] Hoppenheit A. Murugaiyan J. Bauer B. Steuber S. Clausen P. H. Roesler U. (2013). Identification of Tsetse (*Glossina* spp.) using matrix-assisted laser desorption/ionisation time of flight mass spectrometry. PLoS Negl. Trop. Dis. 7:e2305. 10.1371/journal.pntd.0002305 23875040 PMC3708848

[B27] Hung G. C. Chilton N. B. Beveridge I. Gasser R. B. (2000). A molecular systematic framework for equine strongyles based on ribosomal DNA sequence data. Int. J. Parasitol. 30, 95–103. 10.1016/S0020-7519(99)00166-6 10675750

[B28] Hung G. C. Chilton N. B. Beveridge I. McDonnell A. Lichtenfels J. R. Gasser R. B. (1997). Molecular delineation of *Cylicocyclus nassatus* and *C. ashworthi* (Nematoda: Strongylidae). Int. J. Parasitol. 27, 601–605. 10.1016/S0020-7519(96)00192-0 9193955

[B29] Hung G. C. Chilton N. B. Beveridge I. Zhu X. Q. Lichtenfels J. R. Gasser R. B. (1999a). Molecular evidence for cryptic species within *Cylicostephanus minutus* (Nematoda: Strongylidae). Int. J. Parasitol. 29, 285–291. 10.1016/S0020-7519(98)00203-3 10221629

[B30] Hung G. C. Gasser R. B. Beveridge I. Chilton N. B. (1999b). Species-specific amplification by PCR of ribosomal DNA from some equine strongyles. Parasitol. 119(Pt 1), 69–80. 10446706 10.1017/s0031182099004497

[B31] Ionita M. Howe D. K. Lyons E. T. Tolliver S. C. Kaplan R. M. Mitrea I. L.. (2010). Use of a reverse line blot assay to survey small strongyle (Strongylida: Cyathostominae) populations in horses before and after treatment with ivermectin. Vet. Parasitol. 168, 332–337. 10.1016/j.vetpar.2009.11.021 20045254

[B32] Kaplan R. M. (2002). Anthelmintic resistance in nematodes of horses. Vet. Res. 33, 491–507. 10.1051/vetres:2002035 12387486

[B33] Karger A. Kampen H. Bettin B. Dautel H. Ziller M. Hoffmann B.. (2012). Species determination and characterization of developmental stages of ticks by whole-animal matrix-assisted laser desorption/ionization mass spectrometry. Ticks Tick Borne Dis. 3, 78–89. 10.1016/j.ttbdis.2011.11.002 22487425

[B34] Kaye J. N. Love S. Lichtenfels J. R. McKeand J. B. (1998). Comparative sequence analysis of the intergenic spacer region of cyathostome species. Int. J. Parasitol. 28, 831–836. 10.1016/S0020-7519(98)00031-9 9650064

[B35] Kharchenko V. Kuzmina T. Trawford A. Getachew M. Feseha G. (2009). Morphology and diagnosis of some fourth-stage larvae of cyathostomines (Nematoda: Strongyloidea) in donkeys *Equus asinus* L. from Ethiopia. Syst. Parasitol. 72, 1–13. 10.1007/s11230-008-9152-8 19048404

[B36] Kooyman F. N. J. van Doorn D. C. K. Geurden T. Wagenaar J. A. (2016). Semi-quantitative differentiation of cyathostomin larval cultures by reverse line blot. Vet. Parasitol. 216, 59–65. 10.1016/j.vetpar.2015.12.009 26801596

[B37] Kornaś S. Gawor J. Cabaret J. Molenda K. Skalska M. Nowosad B. (2009). Morphometric identification of equid cyathostome (Nematoda: Cyathostominae) infective larvae. Vet. Parasitol. 162, 290–294. 10.1016/j.vetpar.2009.03.018 19359100

[B38] Kuzmina T. A. Kharchenko V. A. Starovir A. I. Dvojnos G. M. (2005). Analysis of the strongylid nematodes (Nematoda: Strongylidae) community after deworming of brood horses in Ukraine. Vet. Parasitol. 131, 283–290. 10.1016/j.vetpar.2005.05.010 15979240

[B39] Kuzmina T. A. Kharchenko V. O. (2008). Anthelmintic resistance in cyathostomins of brood horses in Ukraine and influence of anthelmintic treatments on strongylid community structure. Vet. Parasitol. 154, 277–288. 10.1016/j.vetpar.2008.03.024 18485600

[B40] Lichtenfels J. R. (1975). Helminths of Domestic Equids. Illustrated keys to genera and species with emphasis on North American forms. Proc. Helminthol. Soc. Wash. 42, 1–83.

[B41] Lichtenfels J. R. (2008). Preface. Vet. Parasitol. 156, 1–3. 10.1016/j.vetpar.2008.06.026 18692965

[B42] Lichtenfels J. R. Gibbons L. M. Krecek R. C. (2002). Recommended terminology and advances in the systematics of the Cyathostominea (Nematoda: Strongyloidea) of horses. Vet. Parasitol. 107, 337–342. 10.1016/S0304-4017(02)00167-X 12163244

[B43] Lichtenfels J. R. Kharchenko V. A. Dvojnos G. M. (2008). Illustrated identification keys to strongylid parasites (Strongylidae: Nematoda) of horses, zebras and asses (Equidae). Vet. Parasitol. 156, 4–161. 10.1016/j.vetpar.2008.04.026 18603375

[B44] Lichtenfels J. R. Kharchenko V. A. Krecek R. C. Gibbons L. M. (1998). An annotated checklist by genus and species of 93 species level names for 51 recognized species of small strongyles (Nematoda: Strongyloidea: Cyathostominea) of horses, asses and zebras of the world. Vet. Parasitol. 79, 65–79. 10.1016/S0304-4017(98)00149-6 9777726

[B45] Long E. O. Dawid I. B. (1980). Repeated genes in eukaryotes. Annu. Rev. Biochem. 49, 727–764. 10.1146/annurev.bi.49.070180.003455 6996571

[B46] Loos A. (1900). Notizen zur Helminthologie Egyptens. III. Die Sclerostomen der Pferde und Esel in Egypten. Cent. Bakteriol. 1, 150–160–192.

[B47] Love S. Murphy D. Mellor D. (1999). Pathogenicity of cyathostome infection. Vet. Parasitol. 85, 113–122. 10.1016/S0304-4017(99)00092-8 10485358

[B48] Lyons E. T. Tolliver S. C. Collins S. S. (2007). Study (1991 to 2001) of drug-resistant Population B small strongyles in critical tests in horses in Kentucky at the termination of a 40-year investigation. Parasitol. Res. 101, 689–701. 10.1007/s00436-007-0535-6 17468973

[B49] Lyons E. T. Tolliver S. C. Drudge J. (1999). Historical perspective of cyathostomes: prevalence, treatment and control programs. Vet. Parasitol. 85, 97–112. 10.1016/S0304-4017(99)00091-6 10485357

[B50] Lyons E. T. Tolliver S. C. Kuzmina T. A. Collins S. S. (2010). Critical tests evaluating efficacy of moxidectin against small strongyles in horses from a herd for which reduced activity had been found in field tests in Central Kentucky. Parasitol. Res. 107, 1495–1498. 10.1007/s00436-010-2025-5 20714749

[B51] Matthews J. B. (2014). Anthelmintic resistance in equine nematodes. Int. J. Parasitol. Drugs Drug Resis. 4, 310–315. 10.1016/j.ijpddr.2014.10.003 25516842 PMC4266799

[B52] Mayer-Scholl A. Murugaiyan J. Neumann J. Bahn P. Reckinger S. Nöckler K. (2016). Rapid identification of the foodborne pathogen Trichinella spp. by matrix-assisted laser desorption/ionization mass spectrometry. PLoS ONE 11:e0152062. 10.1371/journal.pone.0152062 26999436 PMC4801418

[B53] McDonnell A. Love S. Tait A. Lichtenfels J. R. Matthews J. B. (2000). Phylogenetic analysis of partial mitochondrial cytochrome oxidase c subunit I and large ribosomal RNA sequences and nuclear internal transcribed spacer I sequences from species of Cyathostominae and Strongylinae (Nematoda, Order Strongylida), parasites of the horse. Parasitology 121, 649–659. 10.1017/s003118200000696x 11155936

[B54] McWilliam H. E. G. Nisbet A. J. Dowdall S. M. J. Hodgkinson J. E. Matthews J. B. (2010). Identification and characterisation of an immunodiagnostic marker for cyathostomin developing stage larvae. Int. J. Parasitol. 40, 265–275. 10.1016/j.ijpara.2009.08.004 19703459

[B55] Mitchell M. C. Tzelos T. Handel I. McWilliam H. E. G. Hodgkinson J. E. Nisbet A. J.. (2016). Development of a recombinant protein-based ELISA for diagnosis of larval cyathostomin infection. Parasitology 143, 1055–1066. 10.1017/S0031182016000627 27174468

[B56] Molin R. (1861). Il sottordine degli acrofalli ordinato scientificamente secondo i risultamenti delle indagini anatomiche ed embrigeniche. Mem. R. Ist. Veneto Sc. Lett Arti 9, 427–633.

[B57] Murphy D. Love S. (1997). The pathogenic effects of experimental cyathostome infections in ponies. Vet. Parasitol. 70, 99–110. 10.1016/S0304-4017(96)01153-3 9195714

[B58] Nielsen M. K. Reinemeyer C. R. Donecker J. M. Leathwick D. M. Marchiondo A. A. Kaplan R. M. (2014). Anthelmintic resistance in equine parasites-Current evidence and knowledge gaps. Vet. Parasitol. 204, 55–63. 10.1016/j.vetpar.2013.11.030 24433852

[B59] Osterman Lind E. Eysker M. Nilsson O. Uggla A. Höglund J. (2003). Expulsion of small strongyle nematodes (cyathostomin spp) following deworming of horses on a stud farm in Sweden. Vet. Parasitol. 115, 289–299. 10.1016/S0304-4017(03)00200-0 12944042

[B60] Perera M. R. Vanstone V. A. Jones M. G. (2005). A novel approach to identify plant parasitic nematodes using matrix-assisted laser desorption/ionization time-of-flight mass spectrometry. Rapid Commun. Mass Spectrom. 19, 1454–1460. 10.1002/rcm.1943 15880621

[B61] Perera M. Taylor S. Jones M. Vanstone V. (2009). Protein biomarkers to distinguish oat and lucerne races of the stem nematode, Ditylenchus dipsaci, with quarantine significance for Western Australia. Nematology 11, 555–563. 10.1163/138855409X12465362560557

[B62] Santos D. W. Dias de Castro L. L. Giese E. G. Molento M. B. (2016). Morphometric study of infective larvae of cyathostomins of horses and their distribution. J. Equine Vet. Sci. 44, 49–53. 10.1016/j.jevs.2016.02.237

[B63] Singhal N. Kumar M. Virdi J. S. (2016). MALDI-TOF MS in clinical parasitology: applications, constraints and prospects. Parasitology 143, 1491–1500. 10.1017/S0031182016001189 27387025

[B64] Smyth J. D. (1990). In Vitro Cultivation of Parasitic Helminths. Boca Raton, FL: CRC Press Inc.

[B65] Tolliver S. C. (2000). A Practical Method of Identification of the North American Cyathostomes (Small Strongyles) in Equid in Kentucky. Lexington: University of Kentucky, Department of Veterinary Science.

[B66] Traversa D. Iorio R. Klei T. R. Kharchenko V. A. Gawor J. Otranto D.. (2007). New method for simultaneous species-specific identification of equine strongyles (nematoda, strongylida) by reverse line blot hybridization. J. Clin. Microbiol. 45, 2937–2942. 10.1128/JCM.00714-07 17626168 PMC2045237

[B67] Traversa D. Kuzmina T. Kharchenko V. A. Iorio R. Klei T. R. Otranto D. (2008). Haplotypic variability within the mitochondrial gene encoding for the cytochrome c oxidase 1 (cox1) of *Cylicocyclus nassatus* (Nematoda, Strongylida): evidence for an affiliation between parasitic populations and domestic and wild equid hosts. Vet. Parasitol. 156, 241–247. 10.1016/j.vetpar.2008.05.031 18619736

[B68] Traversa D. Milillo P. Barnes H. von Samson-Himmelstjerna G. Schurmann S. Demeler J.. (2010). Distribution and species-specific occurrence of cyathostomins (Nematoda, Strongylida) in naturally infected horses from Italy, United Kingdom and Germany. Vet. Parasitol. 168, 84–92. 10.1016/j.vetpar.2009.10.006 19906489

[B69] Von Samson-Himmelstjerna G. (2012). Anthelmintic resistance in equine parasites - detection, potential clinical relevance and implications for control. Vet. Parasitol. 185, 2–8. 10.1016/j.vetpar.2011.10.010 22100141

[B70] Wieser A. Schneider L. Jung J. Schubert S. (2012). MALDI-TOF MS in microbiological diagnostics-identification of microorganisms and beyond (mini review). Appl. Microbiol. Biotechnol. 93, 965–974. 10.1007/s00253-011-3783-4 22198716

[B71] Yssouf A. Almeras L. Raoult D. Parola P. (2016). Emerging tools for identification of arthropod vectors. Future Microbiol. 11, 549–566. 10.2217/fmb.16.5 27070074

[B72] Yssouf A. Socolovschi C. Flaudrops C. Ndiath M. O. Sougoufara S. Dehecq J. S.. (2013). Matrix-assisted laser desorption ionization - time of flight mass spectrometry: an emerging tool for the rapid identification of mosquito vectors. PLoS ONE 8:e72380. 10.1371/journal.pone.0072380 23977292 PMC3744494

